# Supplementary material for: Elafin as a Prognostic Marker in Esophageal Squamous Cell Carcinoma: A Pilot Study Using Three-Dimensional Imaging and Genomic Profiling
Source: Cancers (Basel). 2023 Jul 27;15(15):3825. doi: 10.3390/cancers15153825 (PMC10417143; doi:10.3390/cancers15153825)

Full unedited gel for Figure 5D

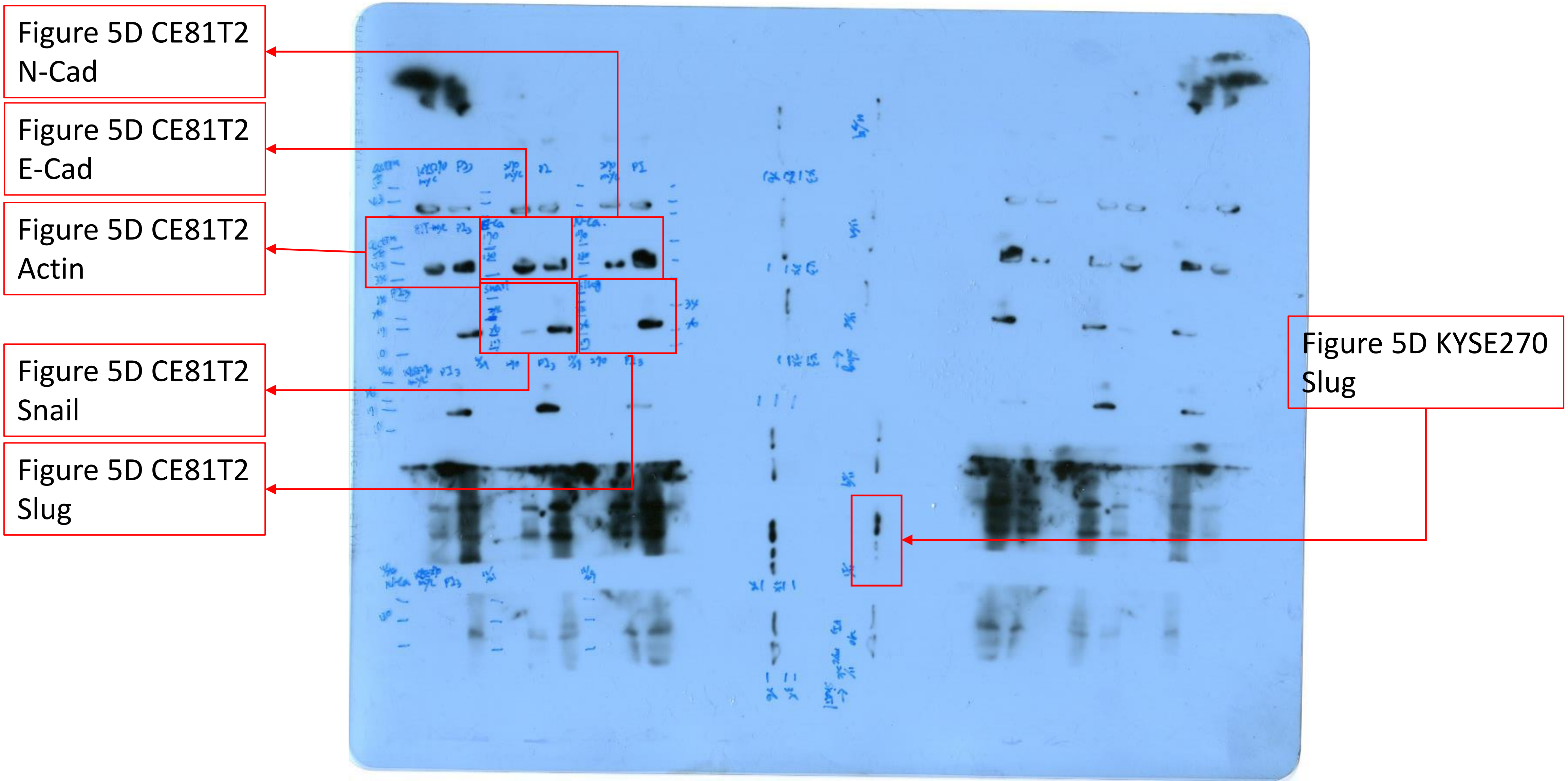

Full unedited gel for Figure 5D

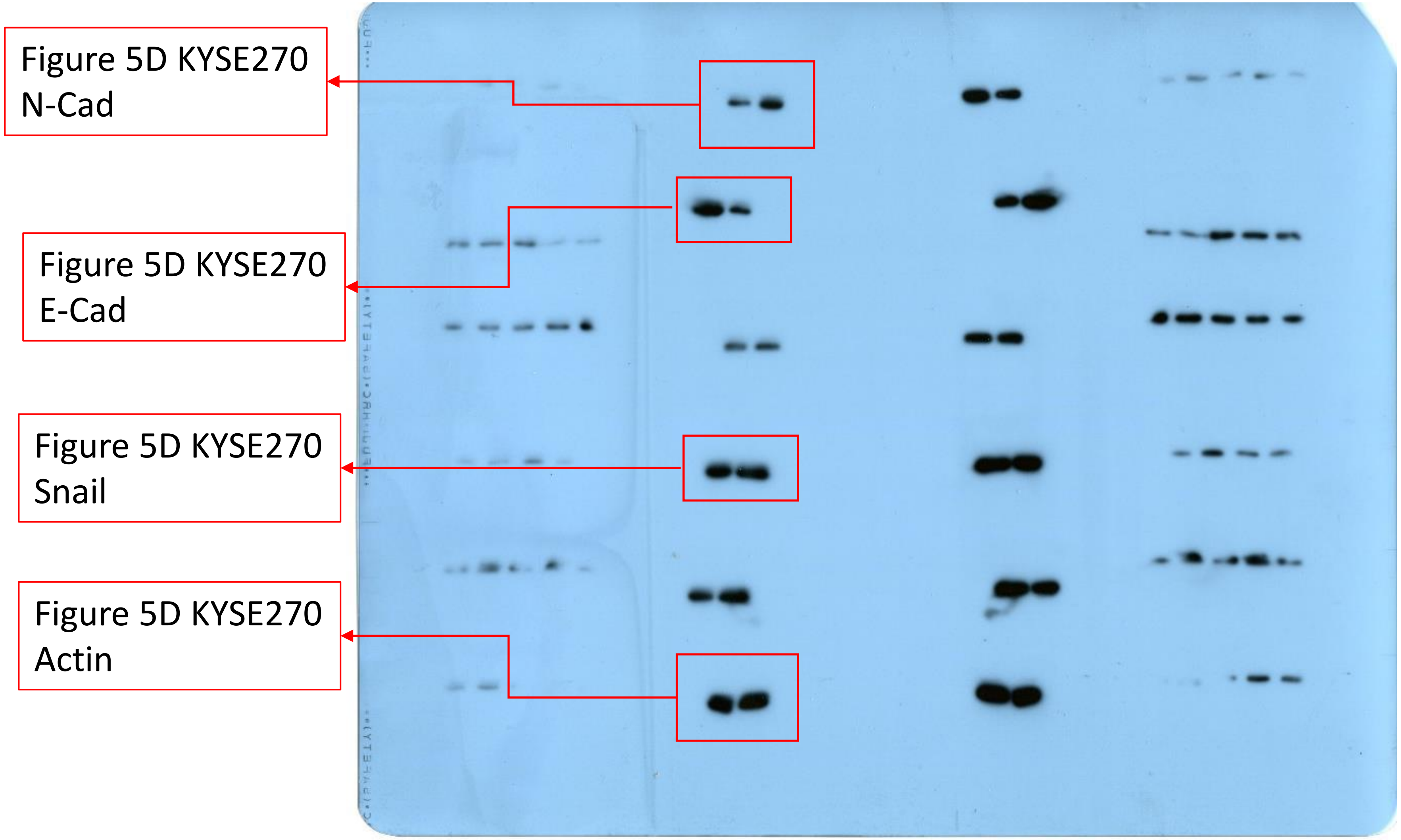

Full unedited gel for Figure 5E

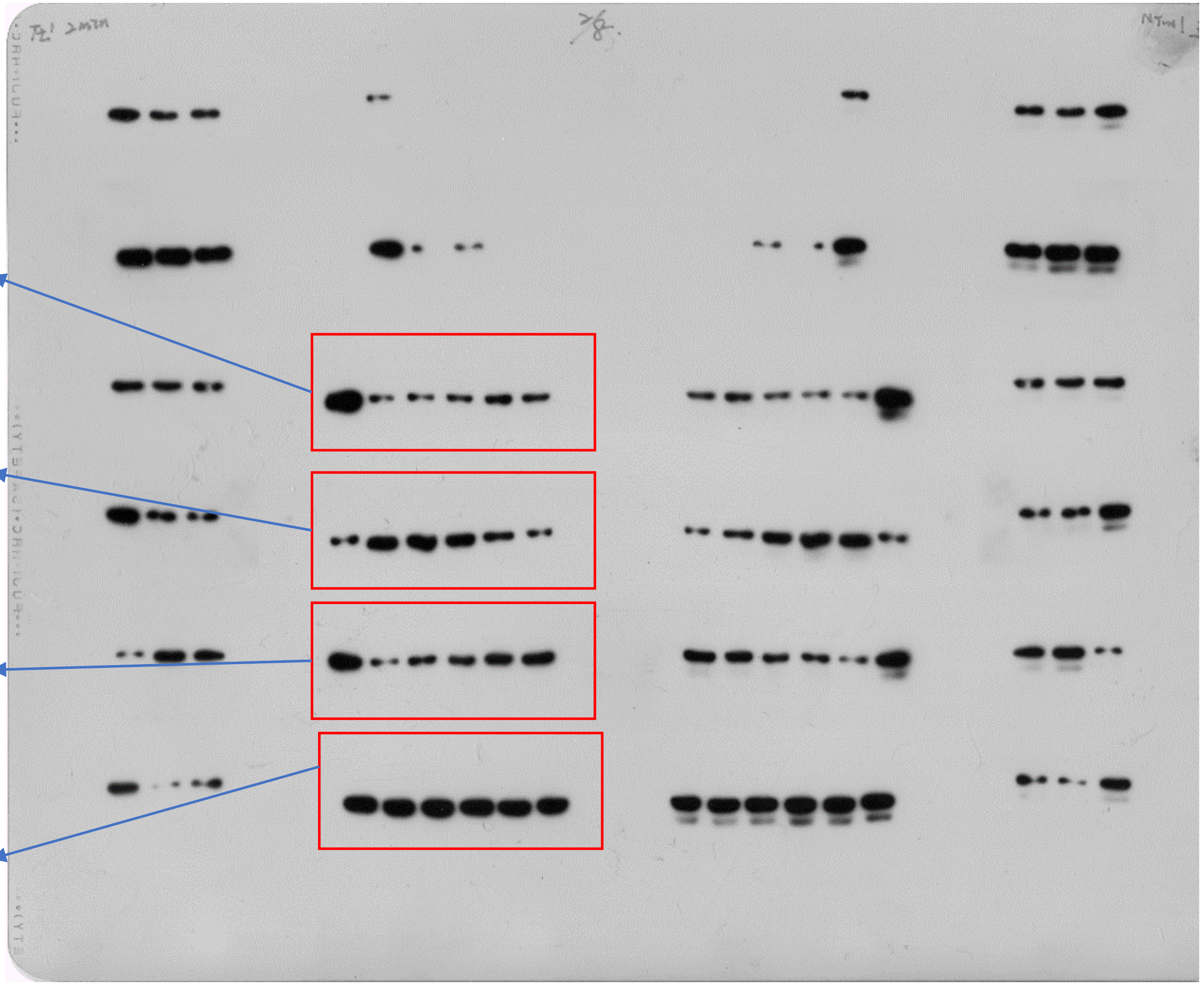

Figure 5E
CE81T2-4_shRNA6
N-Cad

Figure 5E
CE81T2-4_shRNA6
E-Cad

Figure 5E
CE81T2-4_shRNA6
Snail

Figure 5E
CE81T2-4PshRNA6
Actin

Full unedited gel for Figure 5E

Figure 5E
CE81T2-4_shRNA6
Slug

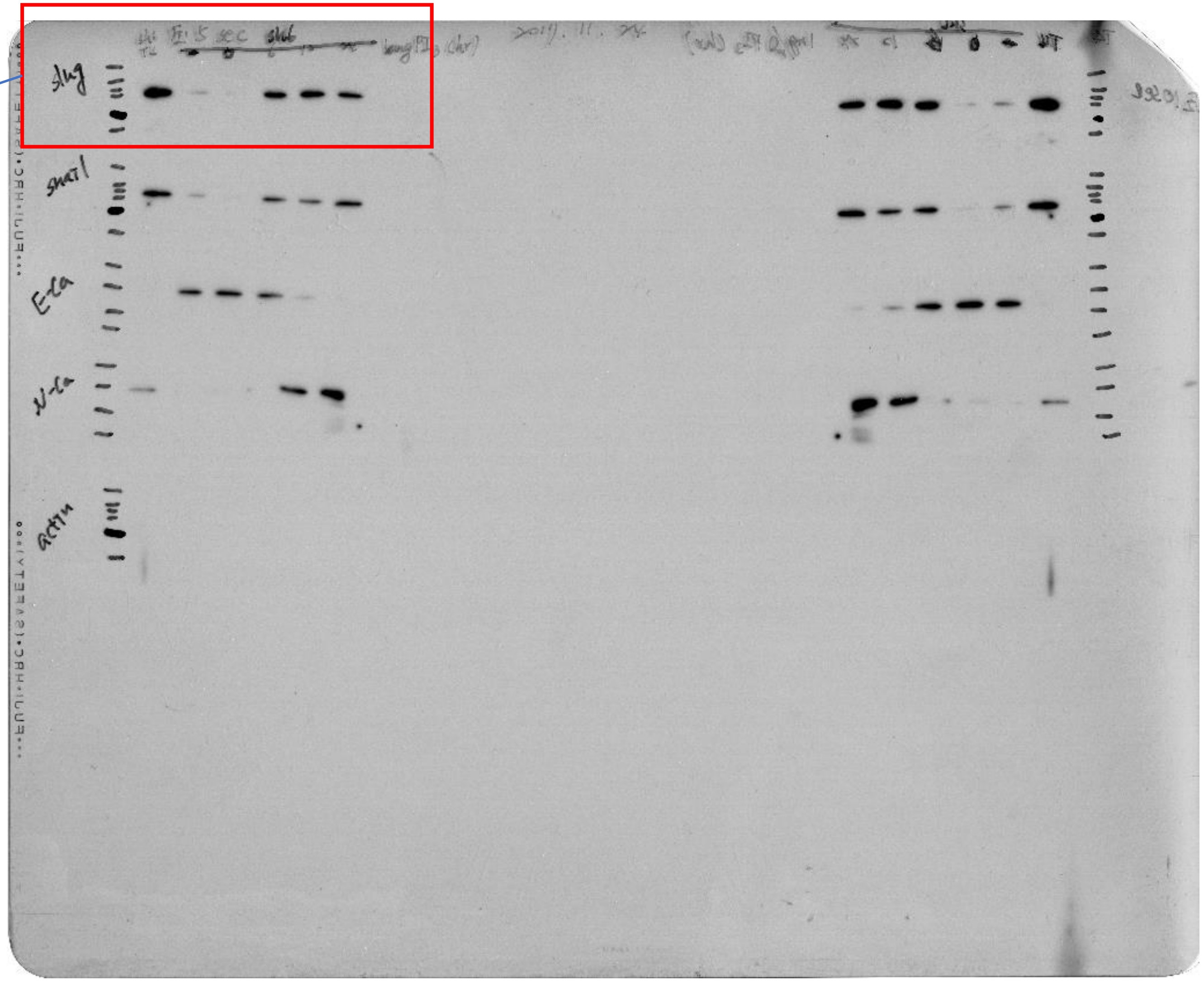

Full unedited gel for Figure 5E

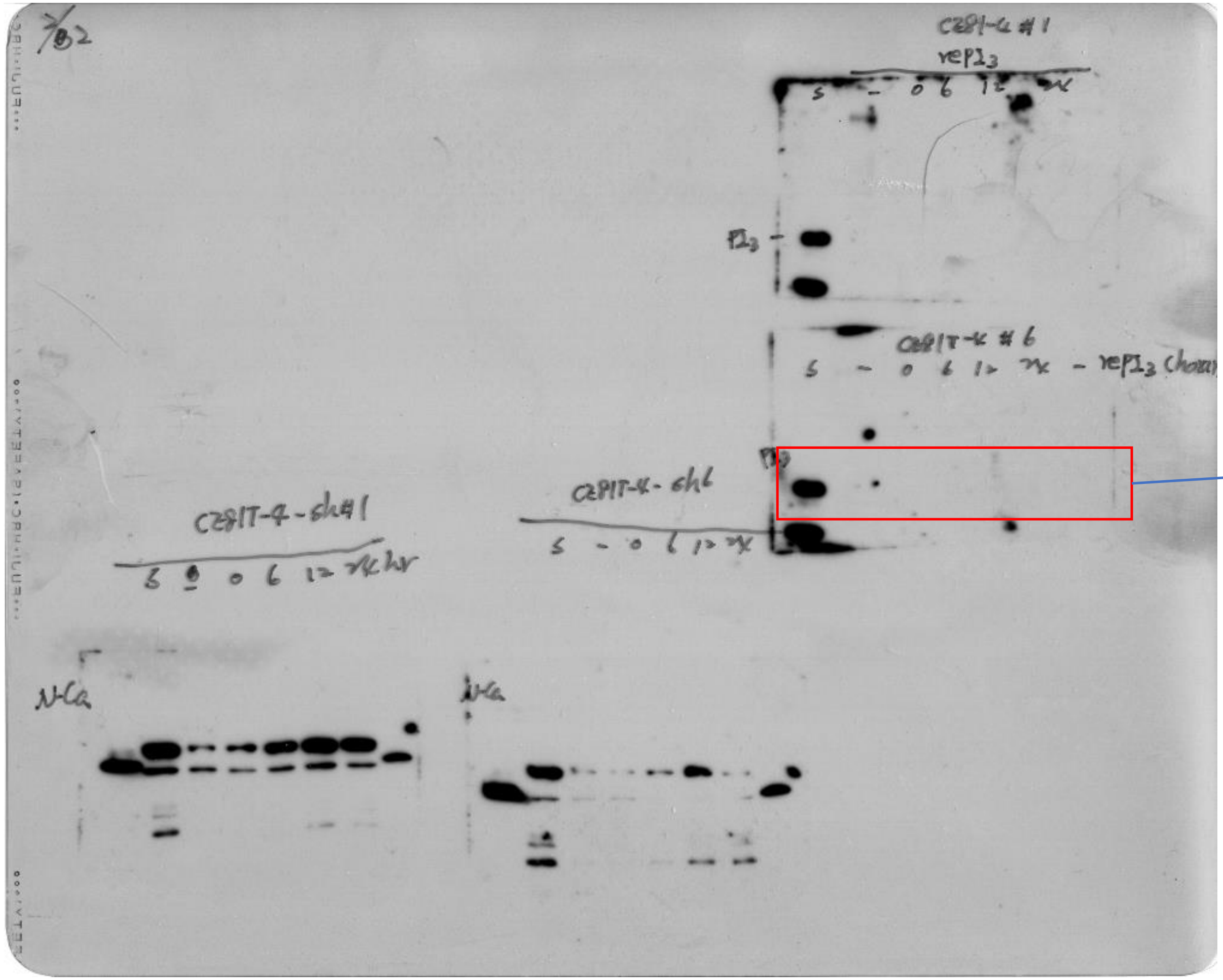

Figure 5E
CE81T2-4_shRNA6
Elafin

Full unedited gel for Figure 5F

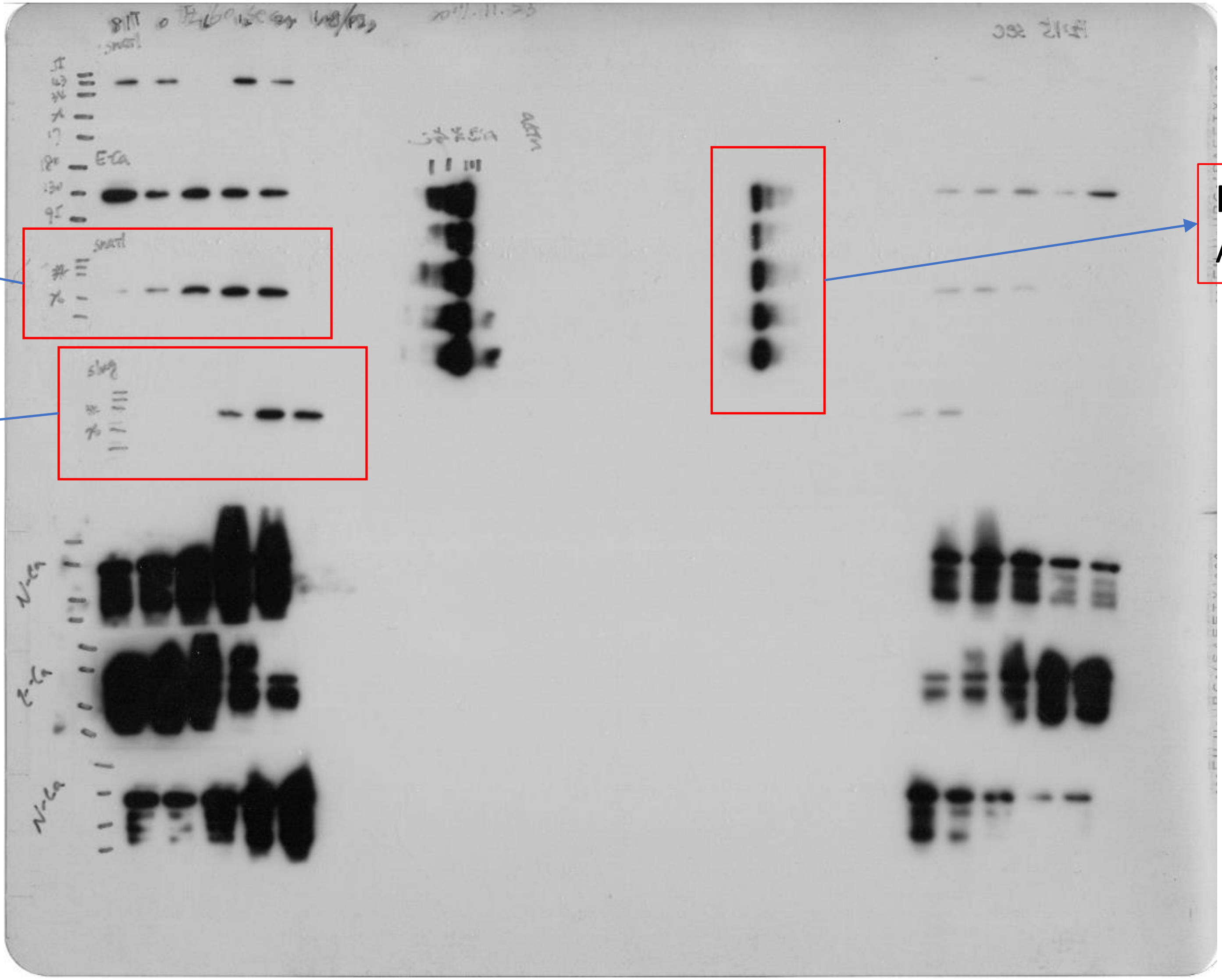

Figure 5F CE81T2 Snail

Figure 5F CE81T2 Slug

Figure 5F CE81T2 Actin

Full unedited gel for Figure 5F

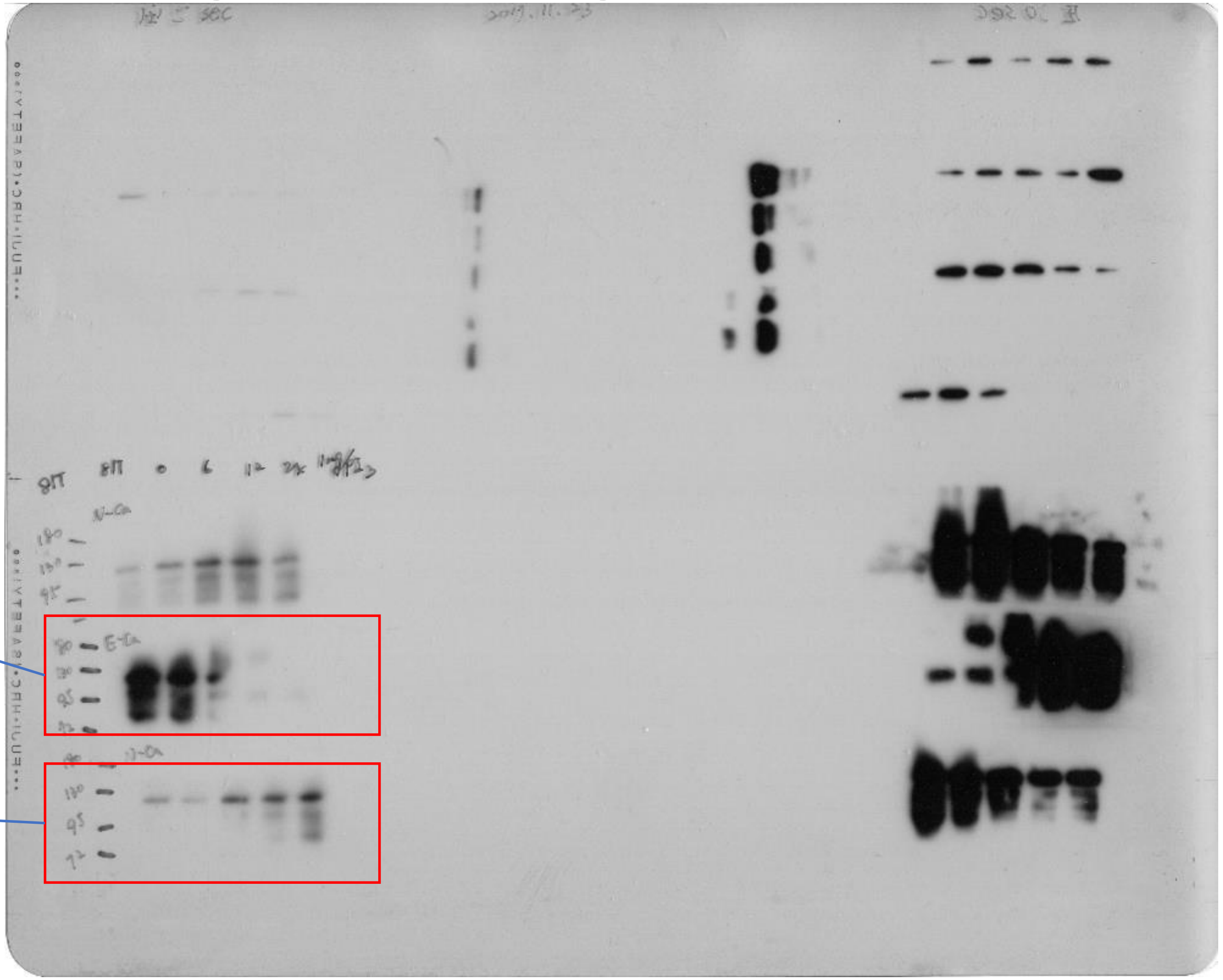

Figure 5F CE81T2 E-Cad

Figure 5F CE81T2 N-Cad

Full unedited gel for Figure S5C

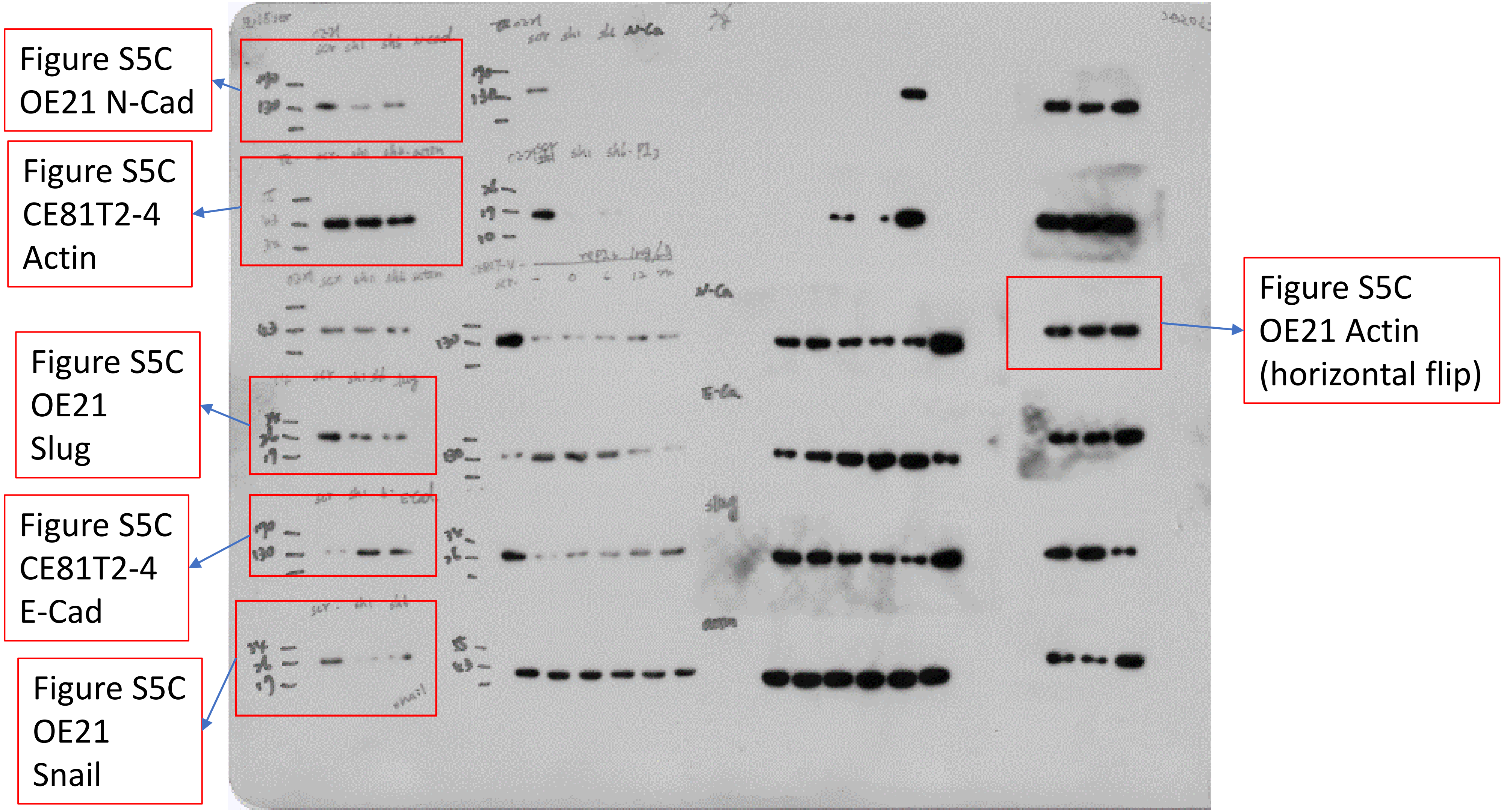

Full unedited gel for Figure S5C

Figure S5C
CE81T2-4
N-Cad

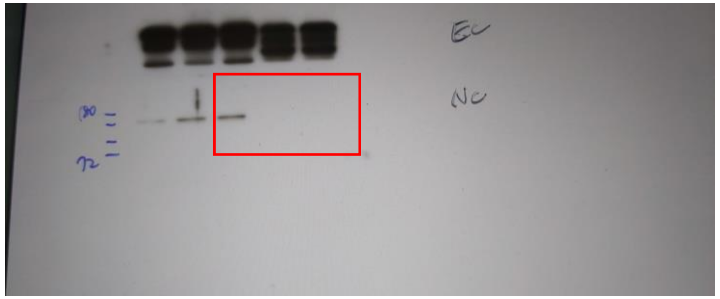

Figure S5C
OE21 E-Cad

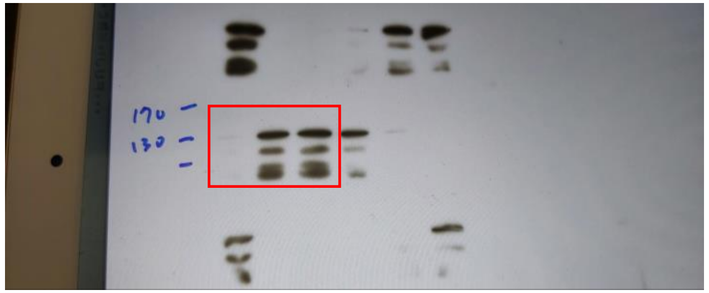

Figure S5C
CE81T2-4
Snail

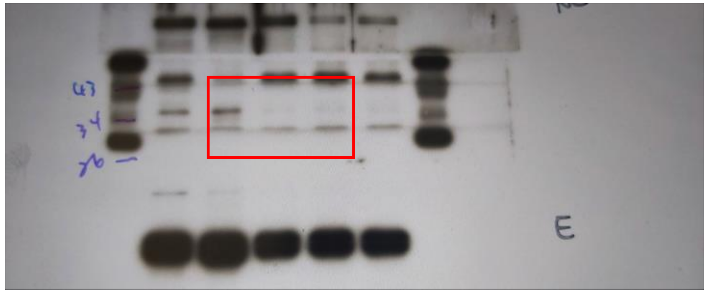

Figure S5C
CE81T2-4
Slug

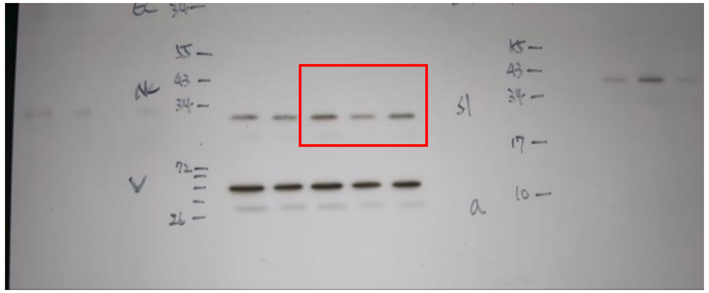

Supplement: Supplementary file 1 [file cancers-15-03825-s001.zip › cancers-2502306-supplementary/cancers-2502306-supplementary File S1.pdf]
