# Supplementary material for: Elafin as a Prognostic Marker in Esophageal Squamous Cell Carcinoma: A Pilot Study Using Three-Dimensional Imaging and Genomic Profiling
Source: Cancers (Basel). 2023 Jul 27;15(15):3825. doi: 10.3390/cancers15153825 (PMC10417143; doi:10.3390/cancers15153825)
Supplement: Supplementary file 1 [file cancers-15-03825-s001.zip › cancers-2502306-supplementary/Cancers_Supplementary Material_revision1.pdf]

Supplementary Material for

Elafin as a Prognostic Marker in Esophageal Squamous Cell Carcinoma: A Pilot Study using 3D Imaging and Genomic Profiling

Wei-Chung Chen[1], Chun-Chieh Wu[2,3], Yu-Peng Liu[4], Guan-Yu Zhuo[5], Yao-Kuang Wang[2,6], Yi-Hsun Chen[6], Chu-Chih Chen[7], Yin-Han Wang[7], Ming-Tsang Wu[1,8], I-Chen Wu[2,6,9]*

This supplementary material includes:

Description of dataset 1

Table S1 to Table S5

Figure S1 to Figure S5

Video S1 to Video S4

References of Table S5

Dataset 1: Whole gene expression of CE81T2-myc (overexpression control), CE81T2-elafin-myc (elafin overexpression), CE81T2-4 scramble control (knock down control), and CE81T2-4-Sh6 (elafin-knock down) by next generation sequencing service of Agilent Technologies.

This database already uploaded on Gene Expression Omnibus database as GSE205623, and will be available to the public once this article is accepted.

Table S1. (A) Number of layers and mean scores of elafin intensity in each cluster of the two comparison pairs. (B) Ranking of the observed mean difference (in red) of the clustered elafin intensity scores among all random reallocations.

(A)

Cluster	Pair 1				Pair 2			
	E365		E385		E535		E421	
	N (% of 1)	Mean	N (% of 1)	Mean	N (% of 1)	Mean	N (% of 1)	Mean
1	9 (100)	1	9 (0)	0	9 (0)	0	10 (0)	0
2	10 (100)	1	9 (0)	0	10 (50)	0.5	11 (0)	0
3	10 (100)	1	9 (0)	0	10 (100)	1	11 (0)	0
4	10 (20)	0.2	9 (0)	0	9 (100)	1	11 (0)	0
5	10 (0)	0	9 (0)	0	10 (100)	1	10 (0)	0
6	10 (0)	0	10 (0)	0	10 (40)	0.4	11 (0)	0
7	10 (0)	0	9 (0)	0	9 (0)	0	11 (0)	0
8	10 (0)	0	9 (0)	0	10 (0)	0	11 (0)	0
9	10 (0)	0	9 (0)	0	10 (0)	0	11 (0)	0
10	10 (0)	0	9 (0)	0	9 (0)	0	10 (0)	0
11	10 (0)	0	9 (0)	0	10 (0)	0	11 (0)	0
12	10 (0)	0	9 (0)	0	10 (0)	0	11 (0)	0
13	10 (0)	0	10 (0)	0	10 (0)	0	11 (0)	0
14	10 (0)	0	9 (0)	0	9 (0)	0	11 (0)	0
15	10 (0)	0	9 (0)	0	10 (0)	0	10 (0)	0
16	10 (0)	0	9 (0)	0	10 (0)	0	11 (0)	0
17	10 (0)	0	9 (0)	0	9 (0)	0	11 (0)	0
18	10 (0)	0	9 (0)	0	10 (0)	0	11 (0)	0
19	10 (0)	0	9 (0)	0	10 (0)	0	11 (0)	0
20	10 (0)	0	9 (0)	0	9 (0)	0	10 (0)	0

(B)

**Pair 1 (E365 vs. E385)**		**Pair 2 (E535 vs. E421)**			
Mean difference	Frequency	Mean difference	Frequency	Mean difference	Frequency
-0.16	65536	-0.195	32768	0.005	98304
-0.14	65536	-0.155	32768	0.045	98304
-0.06	196608	-0.145	32768	0.055	98304
-0.04	196608	-0.105	32768	0.095	98304
0.04	196608	-0.095	98304	0.105	32768
0.06	196608	-0.055	98304	0.145	32768
0.14	65536	-0.045	98304	0.155	32768
0.16	65536	-0.005	98304	0.195	32768

Table S2. Baseline characteristics of 63 locoregional ESCC patients for tissue immunohistochemistry staining of elafin.

**Elafin protein expression in tissue arrays (n = 63)[a]**			**P value**
Male (%)		62 (98.4)	
Age, yrs		57.17±11.62	
Tumor score	0	5 (7.9)	
	1	22 (34.9)	
	1.5	1 (1.6)	
	2	14 (22.2)	
	3	16 (25.4)	
	missing	5 (7.9)	
Normal score	0	3 (4.8)	
	1	26 (41.3)	
	1.5	4 (6.3)	
	2	8 (12.7)	
	missing	22 (34.9)	
		**Mean score (± SD)**	
Normal only (n = 41)		1.17±0.52	
Tumor only (n = 58)		1.72±0.97	**<0.0001[b]**
Difference in paired (T-N) (n = 36)		0.53±1.10	**0.006[c]**

[a]Data are presented as n (%) or mean ± SD.

[b]Wilcoxon rank sum test.

[c]Wilcoxon signed ranked test.

Table S3. Elafin promoted the proliferation of ESCC cell lines.

Elafin manipulation	Doubling time (hours)			
	**CE81T2**	**KYSE270**	**OE21**	**CE81T2-4**
Overexpression				
Myc	24.6±1.5	29.4±1.3	NA	NA
Elafin-myc	20.7±0.7[a]	25.7±0.9[a]	NA	NA
Knockdown				
Scramble	NA	NA	20.5±0.4	21.4±0.3
shElafin #1	NA	NA	26.5±1.4[b]	26.3±0.2[b]
shElafin #6	NA	NA	26.5±2.4[b]	25.6±0.02[b]

NA. not available

[a]*P*<0.05, compared to myc control cells

[b]*P*<0.05, compared to the scramble control cells

Table S4. Gene expression that are related with mesenchymal and epithelial markers after overexpressing in CE81T2 or knockdowning in CE81T2-4.

	Gene symbol	CE81T2_Elafin/ CE81T2_myc (FPKM Log_2 ratio)	CE81T2-4_Sh6/ CE81T2-4_Scr (FPKM Log_2 ratio)
Epithelial markers	FOXA2	0.429599989	0.64291716
	MST1R	0.151935118	0.250063583
	CTNNA1	0.020569151	0.210860369
	PTP4A1	0.022118376	0.132822609
	GSC	0	10.87502744
	KRT32	0	1.137443009
	CDH1	-0.028653927	0.084503857
	LAMA3	-0.025543071	0.062583531
	CTNNB1	-0.128441712	0.352366815
	KRT7	-0.127838992	0.037778945
	FGFBP1	-0.375034632	-0.036013612
Mesenchymal markers	ZEB1	2.818437436	-2.658923843
	NES	0.673191947	-0.742560707
	SPP1	0.44743237	-1.651540783
	SOX9	0.411527454	0.380251678
	CTGF	0.388643493	0.178817974
	TWIST1	0.367803664	0.012048495
	MMP3	0.364003753	-15.21787442
	TMEM132A	0.343728724	0.049315448
	MMP2	0.336531555	0.188936685
	GNG11	0.325871007	-0.704125902
	IGFBP4	0.279090455	-0.606191217
	AHNAK	0.287340771	-0.32698529
	EGFR	0.227689682	-0.005464374
	SPARC	0.205713921	-0.241125458
	CD59	0.20868233	0.03641868
	FN1	0.161171404	-0.946917859
	FOXF1	0.15501339	-0.1450207
	GATA6	0.097544498	-0.498625639
	ZNF703	0.073572496	0.041974501
	ITGAV	0.0634668	-0.064261071
	NUDT13	-0.110979642	-0.326959105
	FGFR2	-0.134549924	-0.292310771

Abbreviation: FPKM= Fragments Per Kilobase of transcript per Million

Table S5. Summary of elafin expressions in different human cancers.

Cancer type	Level (RNA or Protein)	Up or down regulated (Cancer tissue vs. normal tissue)	References
**Arise from squamous epithelium**			
Esophageal cancer	Protein	Up	(1)
Head and neck cancer	Protein	Up	(2)
Lung cancer	Protein	Up	(3)
**Arise from glandular cell**			
Breast cancer	Protein	Up	(4)
	RNA	Up	(5)
	RNA & Protein	Down	(6)
	RNA & Protein	Down	(7)
	RNA & Protein	Down	(8)
	Protein	Down	(9)
Ovarian cancer	RNA & Protein	Up	(10)
	RNA & Protein	Not available	(11)
	RNA & Protein	Up	(12)
Colorectal cancer	RNA & Protein	Not available	(13)
**Others**			
Glioblastoma	RNA & Protein	Up	(14)
	RNA & Protein	Up	(15)
Melanoma	RNA & Protein	Down	(16)

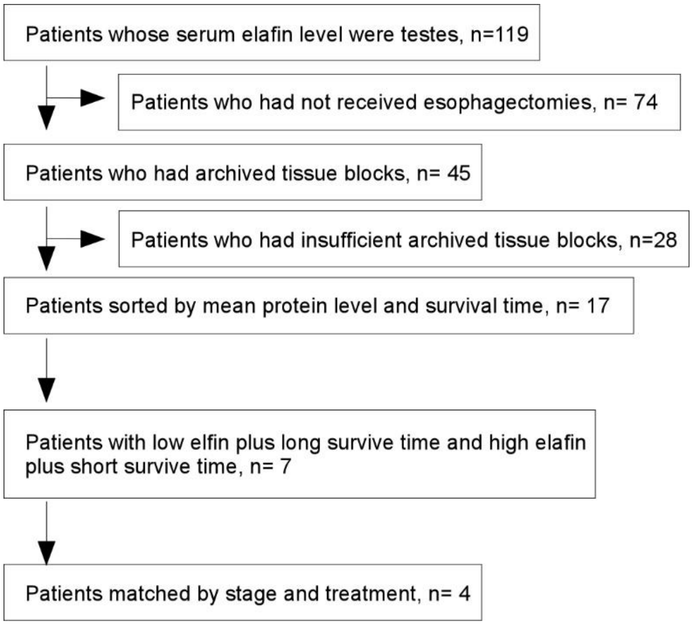

Figure S1. Scheme of patient selection from the 119 ESCC patients.

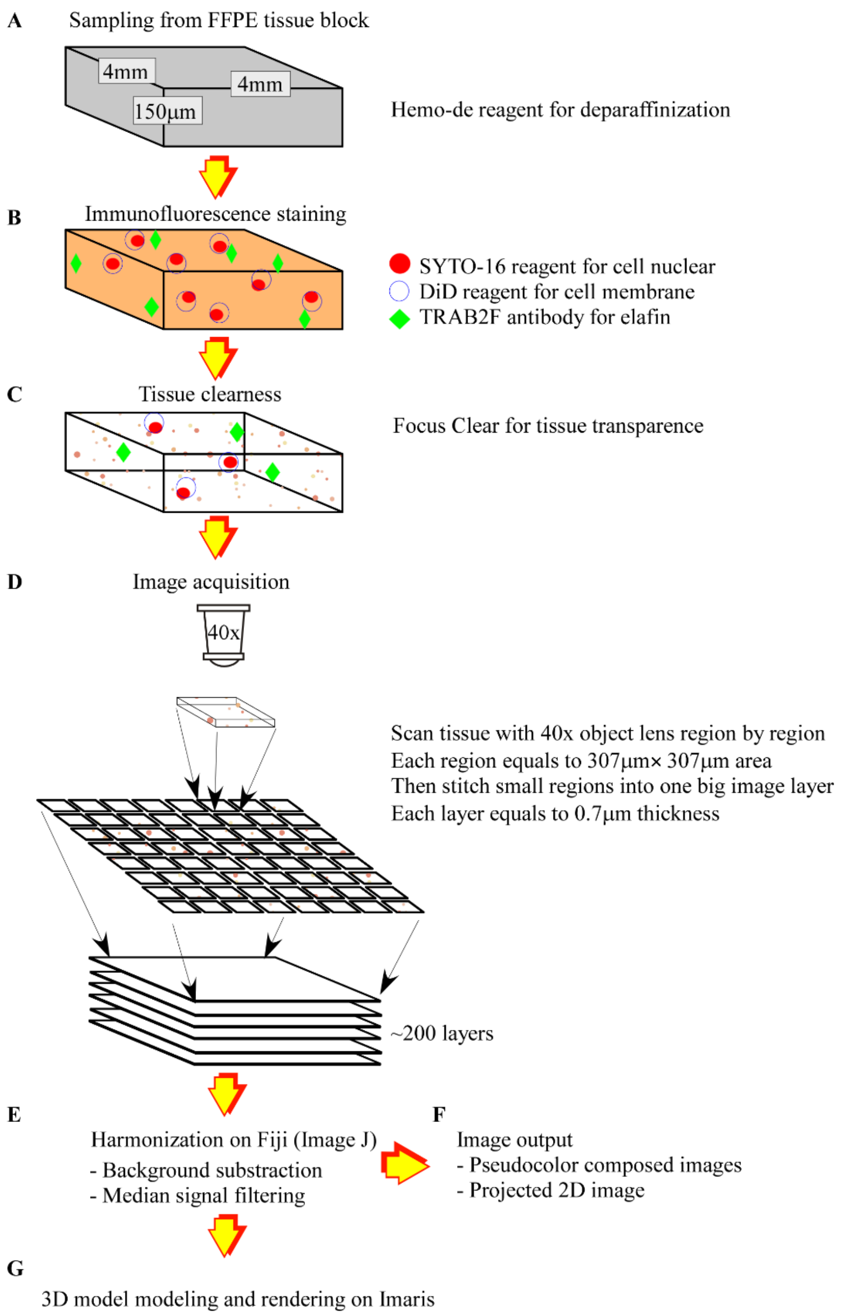

Figure S2. Flowchart of 3D tissue image acquisition, processing and modeling. (A) Matched the HE staining slide with FFPE tissue block and sampled tissue region with biopsy needle. (B) Stained tissue chunk with anti-elafin antibody and two reagents (nuclear and cytoplasm). (C) Tissue cleared by FocusClear solution. (D) Images captured with 40x object lens by confocal microscopy and stitched into one image. The whole tissue specimen was optical sectioned into~200 layers of z-axis. (E) Image harmonized to reduce the noise signal, improve shape of targeted objects, and determine threshold for the construction of 3D architecture. (F) Projected pseudo color image of 200 layers into one layer by Fiji software. (G) Constructed and rendered 3D model of elafin signal and cell nuclear by Imaris software. Abbreviation: FFPE= formalin-fixed, paraffin-embedded

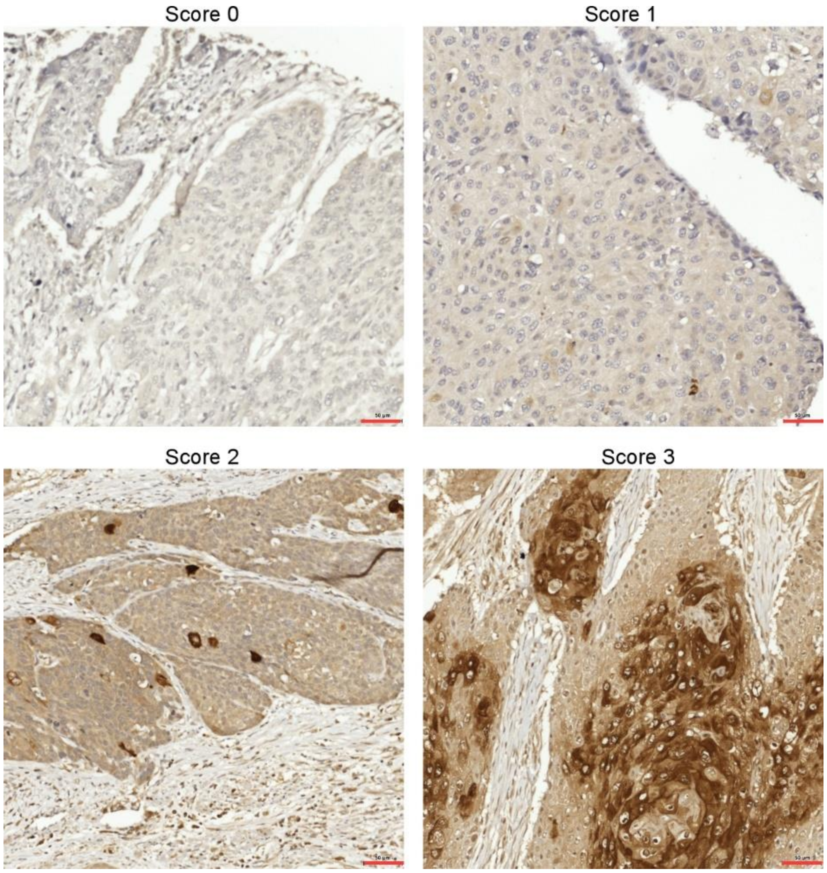

Figure S3. The scoring of elafin staining signal. Score 0 = negative cytoplasmic staining. Score 1 = focal weak cytoplasmic staining. Score 2 = diffuse moderate cytoplasmic staining and blurred membrane staining. Score 3 = diffuse strong cytoplasmic stain and distinct membrane staining. Immunohistochemistry was performed by elafin antibody on a tissue microarray. The representative images show the scoring of elafin signal mentioned in Materials and Methods. Scale bar on bottom right=50μm.

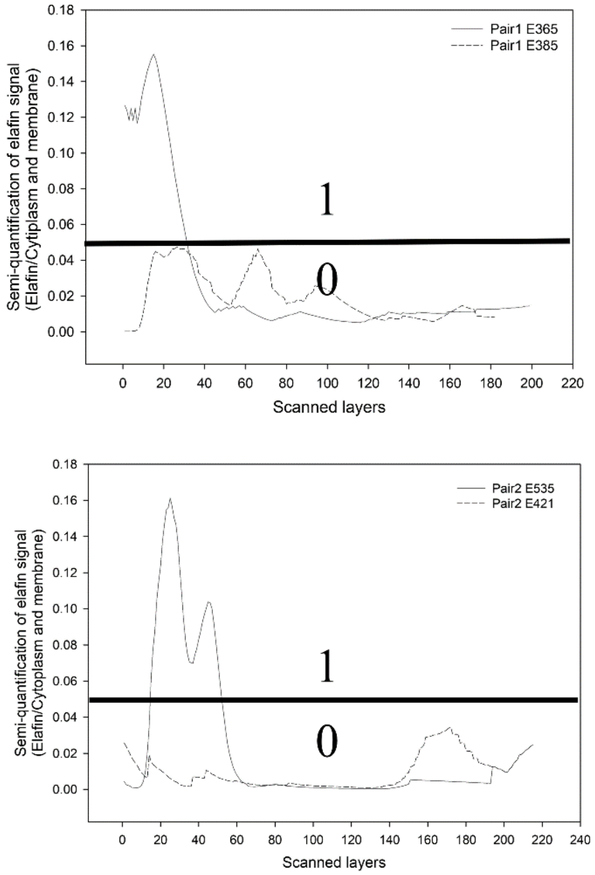

Figure S4. Dichotomous score of the elafin intensities of the tumor vs. normal tissues of the two comparison pairs.

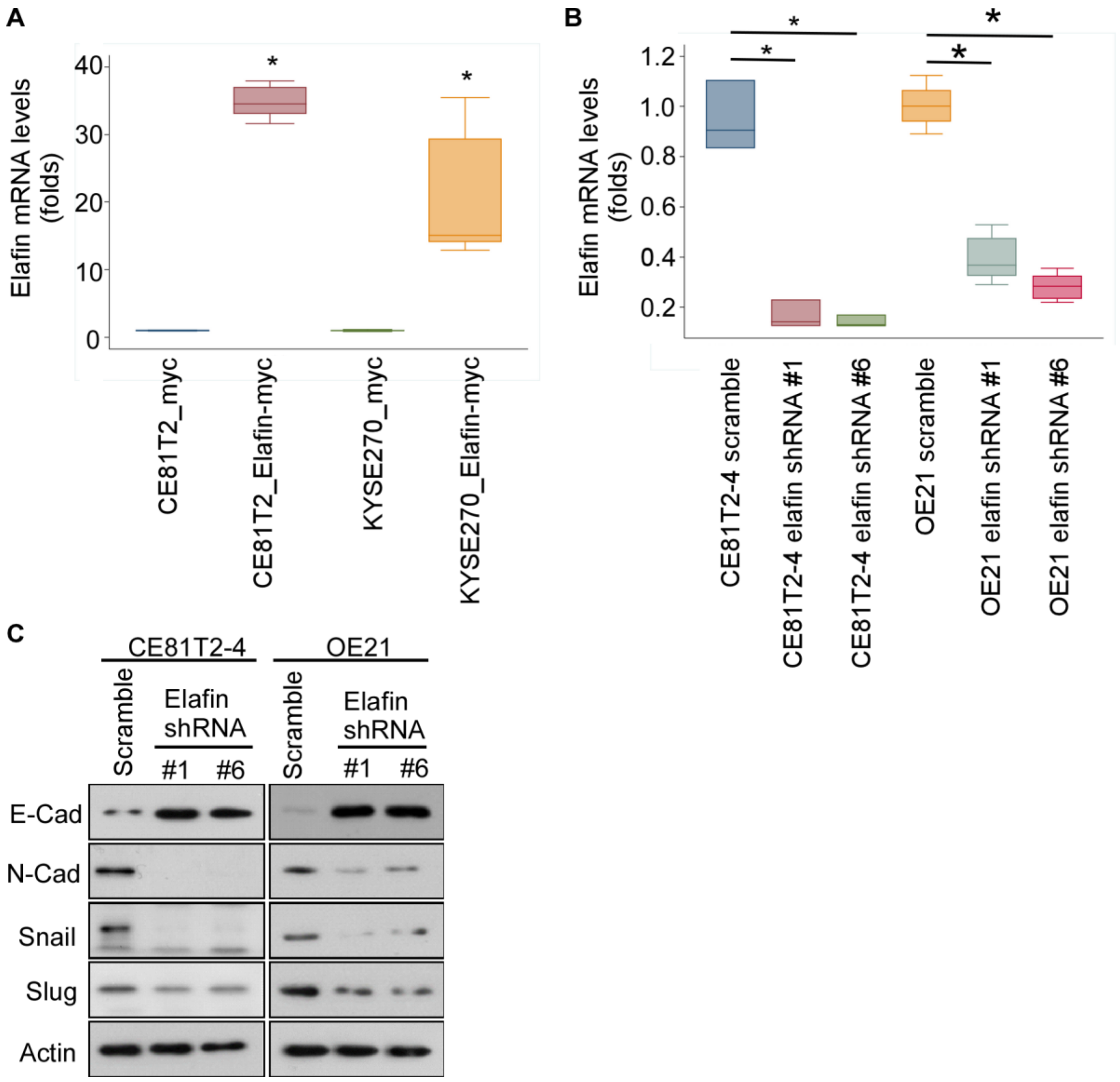

Figure S5. Elafin mRNA levels after transfecting with elafin-expression vector or elafin shRNAs in cell lines with different endogenous elafin levels. (a) Elafin mRNA expression levels increased in the CE81T2 and KYSE270 cell lines after transfecting with ectopic elafin. (b) Elafin mRNA levels decreased in the CE81T2-4 and OE21 cells after being treated with elafin shRNAs. *P<0.05, compared to the controls. (c) Knockdown elafin by elafin-specific shRNAs altered EMT markers in CE81T2-4 and OE21 cell lines

Supplementary Videos

Video S1. The 3-D cuboid of yellow square in figure 2A (E365). The pseudo color green is for elafin, blue for cell membrane and cytoplasm, and red for cell nuclei. Scale bar = 30 μm

Video S2. The 3-D cuboid of yellow square in figure 2G (E535). The pseudo color green is for elafin, blue for cell membrane and cytoplasm, and red for cell nuclei. Scale bar = 30 μm

Video S3. The 3-D cuboid of yellow square in figure 2D (E385). The pseudo color green is for elafin, blue for cell membrane and cytoplasm, and red for cell nuclei. Scale bar = 30 μm

Video S4. The 3-D cuboid of yellow square in figure 2J (E421). The pseudo color green is for elafin, blue for cell membrane and cytoplasm, and red for cell nuclei. Scale bar = 30 μm

Supplementary References

1. Yamamoto S, Egami H, Kurizaki T, et al. Immunohistochemical expression of SKALP/elafin in squamous cell carcinoma of the oesophagus. *Br J Cancer* 1997; **76**(8): 1081-6.

2. Westin U, Nystrom M, Ljungcrantz I, Eriksson B, Ohlsson K. The presence of elafin, SLPI, IL1-RA and STNFalpha RI in head and neck squamous cell carcinomas and their relation to the degree of tumour differentiation. *Mediators Inflamm* 2002; **11**(1): 7-12.

3. Yoshida N, Egami H, Yamashita J, et al. Immunohistochemical expression of SKALP/elafin in squamous cell carcinoma of human lung. *Oncol Rep* 2002; **9**(3): 495-501.

4. Kulasingam V, Diamandis EP. Proteomics analysis of conditioned media from three breast cancer cell lines: a mine for biomarkers and therapeutic targets. *Mol Cell Proteomics* 2007; **6**(11): 1997-2011.

5. Yokota T, Bui T, Liu Y, Yi M, Hunt KK, Keyomarsi K. Differential regulation of elafin in normal and tumor-derived mammary epithelial cells is mediated by CCAAT/enhancer binding protein beta. *Cancer Res* 2007; **67**(23): 11272-83.

6. Caruso JA, Hunt KK, Keyomarsi K. The neutrophil elastase inhibitor elafin triggers rb-mediated growth arrest and caspase-dependent apoptosis in breast cancer. *Cancer Res* 2010; **70**(18): 7125-36.

7. Hunt KK, Wingate H, Yokota T, et al. Elafin, an inhibitor of elastase, is a prognostic indicator in breast cancer. *Breast Cancer Res* 2013; **15**(1): R3.

8. Caruso JA, Karakas C, Zhang J, et al. Elafin is downregulated during breast and ovarian tumorigenesis but its residual expression predicts recurrence. *Breast Cancer Res* 2014; **16**(6): 3417.

9. Caruso JA, Akli S, Pageon L, Hunt KK, Keyomarsi K. The serine protease inhibitor elafin maintains normal growth control by opposing the mitogenic effects of neutrophil elastase. *Oncogene* 2015; **34**(27): 3556-67.

10. Clauss A, Ng V, Liu J, et al. Overexpression of elafin in ovarian carcinoma is driven by genomic gains and activation of the nuclear factor kappaB pathway and is associated with poor overall survival. *Neoplasia* 2010; **12**(2): 161-72.

11. Wei H, Hellstrom KE, Hellstrom I. Elafin selectively regulates the sensitivity of ovarian cancer cells to genotoxic drug-induced apoptosis. *Gynecol Oncol* 2012; **125**(3): 727-33.

12. Labidi-Galy SI, Clauss A, Ng V, et al. Elafin drives poor outcome in high-grade serous ovarian cancers and basal-like breast tumors. *Oncogene* 2015; **34**(3): 373-83.

13. Liu Y, Tian Y, Wu T, Dai Y, Wang W, Teng G. High Expression and Clinical Significance of Elafin in Colorectal Cancer. *Gastroenterol Res Pract* 2019; **2019**: 4946824.

14. Saidi A, Javerzat S, Bellahcene A, et al. Experimental anti-angiogenesis causes upregulation of genes associated with poor survival in glioblastoma. *Int J Cancer* 2008; **122**(10): 2187-98.

15. Verbovsek U, Motaln H, Rotter A, et al. Expression analysis of all protease genes reveals cathepsin K to be overexpressed in glioblastoma. *PLoS One* 2014; **9**(10): e111819.

16. Yu KS, Lee Y, Kim CM, et al. The protease inhibitor, elafin, induces p53-dependent apoptosis in human melanoma cells. *Int J Cancer* 2010; **127**(6): 1308-20.
